# Mutant metaphors: *Frankenstein* in the era of COVID-19

**DOI:** 10.1136/medhum-2022-012405

**Published:** 2022-12-07

**Authors:** Allison Coffelt, Alexandre Djandji

**Affiliations:** 1 Narrative Medicine, Columbia University, New York, New York, USA; 2 Columbia University Fertility Center, Columbia University, New York, New York, USA

**Keywords:** Medical humanities, COVID-19, Health policy, literature and medicine, narrative medicine

## Abstract

Since its debut, Mary Shelley’s *Frankenstein* has, fittingly, assumed a life of its own. In today’s cultural landscape, the mere mention of ‘mutant’ evokes the language of Othering, including Frankensteinian metaphors, such as those used to describe the omicron variant of SARS-CoV-2. When scientists referred to omicron as a Frankenstein variant, they demonstrated the inherent mutability of the myth—a myth that is crucial in biomedicine. In this article, the authors examine the shifting nature of *Frankenstein* metaphors and consider how they function in what Priscilla Wald refers to as outbreak narratives in the context of the USA’s COVID-19 policies. The authors point to the ready instatement of travel bans as evidence of how such a potent myth is used to create and sell public policy. In response to such xenophobic policies, the authors apply Donna Haraway’s concept of ‘boundary breakdowns’ in order to reimagine relationships with mutancy. They examine how moving past the idea of *mutant is other* in contemporary virus narratives may offer a way to reconfigure our relationships of self and other and move beyond the hegemonic and nativist policies of the present.

## Introduction

‘This is probably the most mutated virus we’d ever seen’, warned Alex Sigal, virologist and lead researcher of the team that identified Omicron, the strain of SARS-CoV-2 that dominated the headlines in late 2021 and early 2022 (Jenkins 2021). Sigal was likely referring to the genetic mutability of the virus—which is to say its changeability. When his team first identified Omicron in late November 2021, they identified over 50 possible mutations in the virus, stated an article in *The Hill*. These mutations, according to the same article, ‘reportedly enhanc[e] [omicron’s] ability to infect people’. Both news and fear of this ‘mutant’ spread swiftly around the globe—and with it came metaphor: ‘The thing is a Frankenstein mix of all the greatest hits’, declared Stephen Hoge, president of Moderna (Mandavilli 2021). Setting aside the idea of the virus as a compilation of popular songs, Sigal and Hoge’s separate identifications of the omicron variant’s mutational profile as Frankensteinian demonstrate the extent to which, to quote Donna Haraway, ‘the figure and the reference have become confused with each other’ (Haraway 2000, 00:05:41). In short, the centuries-old novel has become a metaphor. But a metaphor of what, exactly?

That ‘Frankenstein’ stands in for the creature (not creator) *and* for mutant shows not only how far from the Mary Shelley novel the public narrative of Frankenstein has wandered, but also how deeply it has contributed to what Priscilla Wald identifies as ‘outbreak narratives’: the ‘epidemiological stories’ that influence our ‘accounts of disease emergence across genres and media’ (2008, 3). However, a return to the text of *Frankenstein*, when paired with an understanding of what Haraway calls the ‘boundary breakdowns’ of the late twentieth century, may well offer a wedge with which we can pry open a different understanding of and relationship with the viral narratives that are quickly becoming commonplace in the twenty-first century.

## Frankenstein: mutant and metaphor

**Figure 1:**
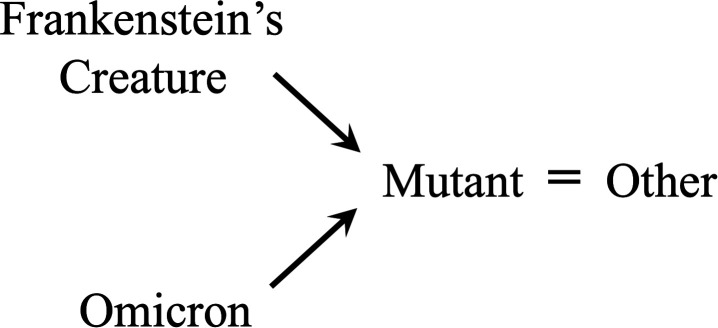
Mutant metaphor formulation.

First, the metaphor of Frankenstein. In *I is an Other: The Secret Life of Metaphor and How it Shapes the Way We See the World*, James Geary offers an at once concise and broad definition. Metaphor, he says, is *x=y* (Geary 2011, 8). In this case, *omicron is mutant*, the equivalency both descriptive and metaphorical. Omicron is in fact a viral mutation, but it is also impossible to separate such a categorisation from the cultural weight of mutancy and its associations—including Other, fear and Frankenstein. In mapping these associations—which overlap more like a collage than line up in an ordered, logical sequence—it is possible to see how it becomes culturally true that omicron is mutant, and with mutant comes fear and Frankenstein and Other (Figure 1).

But *is* the creature from *Frankenstein* a mutant? And perhaps more importantly, is it helpful to equate the omicron variant with the Frankenstenian tale?

Let’s return to the origin story in *Frankenstein*, in which Victor Frankenstein is the creator, not the creation. Having collected severed limbs from the slaughterhouse, brought them to his dissecting room, and worked relentlessly, Dr Frankenstein, on a dreary night in November, ‘beheld the accomplishment of [his] toils’. The ‘dull yellow eye’ of the ‘creature’ opened, and ‘it’ began to breathe (Shelley 1998, 57). Frankenstein goes on to rave:

His limbs were in proportion, and I had selected his features as beautiful. Beautiful!—Great God! His yellow skin scarcely covered the work of muscles and arteries beneath; his hair was of a lustrous black, and flowing; his teeth of pearly whiteness, but these luxuriances only formed a more horrid contrast with his watery eyes, that seemed almost the same colour as the dun white sockets in which they were set, his shriveled complexion and straight black lips. (Shelley 1998, 57)

This creature, who will later learn French and read Milton and long for love (thus signalling his sophistication to the novel’s readership) is more amalgamation than variation. If the definition of a mutant in biology is ‘an organism, gene, etc., which has undergone or arisen by mutation; a mutated form’ (Oxford English Dictionary 2021), then this creature is not that. Though he comes from one who is (arguably) like him, he is not a spawn of an older generation. Instead, he is a mix (a ‘greatest hits’?) of many parts that come together to make a whole. He is the first (and to his despair, the only) of his kind.

And yet the creature is modelled after a human, using human parts. In this sense, he is mutant. Moreover, Frankenstein’s creature certainly meets the other definition of mutant: one that is ‘originally science fiction’ and is ‘an individual imagined as having arisen by genetic mutation’, especially ‘one with freakish or grossly abnormal anatomy, abilities, etc.’ (Oxford English Dictionary 2021). His ‘horrid contrast’, haunting eyes, and ‘shriveled complexion’ leave no doubt in the mind of Shelley’s reader: we are to find this creature grotesque (1998, 57). Any questions one may have are resolved throughout the novel, when humans who encounter the creature are continually horrified by his appearance.

How fitting, then, that this creature both is and is not a mutant. How appropriate that this pliant definition of ‘mutant’ applies to a myth in which there is common conflation of the maker and his creation vis-à-vis the name, ‘Frankenstein’. And while the cultural metaphor of Frankenstein as mutant relies on a simple, direct formulation, Frankenstein’s creature = mutant = Other (figure 1), a closer look at the origins of the associations show more of overlay than progression, more tangles than order. They are, in short, their own amalgamation—replete with their own gross abnormalities.

## Mutant as other

The discrepancy between text and cultural paradigm says more about the reductive possibilities of contagion narratives than it does about Shelley’s novel. The abundant references to *Frankenstein* in news coverage of omicron and other variants are further evidence for Jon Turney’s argument from the late 1990s: that *Frankenstein* is ‘the governing myth of modern biology’ (Turney 1998, 3)—a myth that reflects ambivalent public attitudes towards science. And it is a myth, it seems, whose only obligation is (fittingly) to shapeshift and resurface, rather than remain true to the source material of Shelley’s novel. In contemporary news articles, omicron as a mutant variant is compared with Frankenstein (by which they usually mean creature), which summons fear. Though one could argue that the evocation of *Frankenstein* may help explain the nature of this variant (as that of a mutation), it would be naïve to assume we could easily divorce this association of mutation, and *Frankenstein*, from alarm. Just as Victor Frankenstein rushed from his room on witnessing his creation, heart full of ‘horror and disgust’ (Shelley 1998, 57), so, too, are we as a collective positioned to fight, fly or freeze in response to the threat of such a Frankenstenian, and therefore grotesque, mutant variant.

Because of this rapid leap from mutant to fear, the possibility of understanding Frankenstein’s creature not in terms of either/or (mutant or not, monster or human), but rather in terms of ‘yes, and’ (mutant *and* non-mutant, person *and* creature) evaporates. Like in the novel, the label of ‘Frankenstein’ becomes a call to arms, a common understanding that any ‘mutant’ ‘creature’ is an Other worthy of not just aversion and caution, but attack. Like many medical metaphors, the word ‘mutant’ inspires certain action. Consider how the idea of mutancy maps onto Susan Sontag’s analysis of the metaphors of cancer—a diagnosis that may well involve a conversation about mutant cells: ‘To describe a phenomenon as a cancer [substitute: mutant] is an incitement to violence. The use of cancer [substitute: mutant] in political discourse encourages fatalism and justifies ‘severe’ measures’ (Sontag 1990, 84). It should come as no surprise, then, that military metaphors are so readily ‘deployed’ in the USA’s COVID-19 ‘response’. The use of ‘front line’, ‘war’ and other language reinforced ‘tropes of villainous, external threats that encroach on the American body politic, even as a very real virus threatens the bodies of US citizens’ (Khan, Iwai, and DasGupta 2021). Evoking *Frankenstein*, in other words, prepares us for a fight. When the message is boiled down to the fact that Omicron is a mutant similar to Frankenstein’s creature, the same fear response is evoked. This ultimately works within a tradition of Othering and nativism that cuts across geographical, political and racial lines.

Consequently, government and institutional forces enact policies that dictate social behaviours on individual and societal levels, including those based more on nativist fear than on fact. On an individual level, we are told to socially distance, wear masks when in contact with others and practice good hand hygiene—all of which have been demonstrated to stop the spread of communicable disease. But what of practices that are not evidence-based? The USA, among other countries, mandated various bans on individuals travelling from ‘high-risk’ countries, for instance, South Africa when the omicron variant first emerged, despite a lack of evidence to support such policies (Shivaram, Bowman, and Diaz 2021). Emphasising the ways viral infection responds to social interaction, with a particular attention to blaming a foreign Other, fosters ‘medicalized nativism’, in which immigrant and foreign groups are stigmatised as a result of their geopolitical relationship to communicable disease (Wald 2008, 8). Nativism, ‘xenophobic nationalism’, (Friedman 2017) is the discrimination against foreigners or immigrant groups in favour of those thought to be native to an area. In the case of viral outbreak, non-native groups who are marked as bringing disease ‘in’ and circulating it are positioned against the interests and well-being of natives. As viruses inevitably spread, other groups and communities become associated with the disease—and depending on their pre-existing condition of power, may well be relegated to the status of Frankenstenian creature, the omicron variant, the Other. This othering reinforces hegemonic structures and reifies political, socioeconomic and racist policies.

Indeed, the pattern of contagion, including the ‘identification of an emerging infection, (which) includes discussion of the global networks throughout which it travels, and chronicles the epidemiological work that ends with its containment’ is what Priscilla Wald calls the outbreak narrative (2008, 2). By deconstructing these patterns in film, literature and reportage, she highlights how the circulation of microbes shapes attitudes and fears, and contributes to social transformation. Even in the face of an outbreak narrative in which medicalised nativism can thrive, Wald argues for the *possibility* that analysing such narratives may help us develop ‘more effective, just, and compassionate responses both to a changing world and to the problems of global health and human welfare’ (2008, 3). If, that is, we are willing to realise the power and potential of such narratives.

How might we begin to analyse outbreak narratives to contribute to more equitable and effective responses? According to Wald, a communicable disease that risks infecting large portions of the population emphasises our own interconnectedness, despite defensive strategies of border closures and isolation. In contagion narratives, the wall never holds; the bucket always leaks. In an epidemic, ‘communicability configure[s] community’ (Wald 2008, 12). In this sense, contagion offers a clear view of our own interconnectedness, how we organise our social structures and the fragility of our social and geographical borders: ‘Contagion was the color of belonging, social as well as biological. The common susceptibility of all people attested to the common bonds of humanity, and the idea of a plague as a great equalizer, affecting rich and poor, worldly and devout, was a regular theme in the literature’ (Wald 2008, 12). Though COVID-19 has in no way impacted people of different classes, races, locations and belief systems equally, Wald is speaking to the prominence of the theme of ‘sickness as equalizer’ in contagion texts. By emphasising the socialisation of the contemporary, globalised world, the outbreak narrative seeks ‘to make the routes of cultural transmission as visible as bacteriologists … had made pathways of disease transmission’ (Wald 2008, 14–15). Like the bacteriologist who reveals the microbes that circulate and infect our bodies, so too must we trace and reveal the invisible web of idea exchange, information and the very ‘relationships that constituted the terms of [our] existence’ (Wald 2008, 14). In this regard, contagion makes the invisible visible by revealing both our biological and relational connectedness across borders, time and space. An analysis of outbreak narratives, at its best, highlights the dissolution of perceived boundaries and borders, creating room to break from the rigid and isolationist thinking that fuels discriminatory responses to contagion.

## Boundary breakdowns: towards a regenerative body

It is this boundary breakdown, and its implications, that Donna Haraway explores, and when applied to contagion narratives, this concept offers a way to realise Wald’s ‘conviction’ that studying outbreak narratives can lead to more ‘effective, just, and compassionate responses’ in global health. Writing ‘The Cyborg Manifesto’ almost four decades before COVID-19 and two decades before Wald, Haraway discusses the breach of (1) The ‘boundary between human and animal’, (2) The boundary of ‘animal-human (organism) and machine’ and (3) Its subset ‘physical and non-physical’ (1985, 10). The parallels to our current pandemic are clear: (1) COVID-19 is a zoological virus spreading across a world in which (2) The distinction between ‘natural and artificial’ is ‘thoroughly ambiguous’ (Haraway 1985, 11) as so many humans increasingly live their lives behind and through screens; meanwhile, (3) Both visible and invisible technologies—and narratives—connect us all.

In the face of these dissolutions, Haraway argues, we have an opportunity to see ourselves as cyborgs—a ‘slightly perverse shift of perspective’ that ‘might better enable us to contest for meanings, as well as other forms of power and pleasure in technologically mediated societies’ (1985, 13). Under this premise, we are positioned to see both ourselves and the world as hybrid; we are both mutant and not, both transmitters and victims of virus. With this in mind, the Patient Zero or ‘super-spreader’ Wald describes (2008, 4) is narratively complicated, rather than easily blamed. ‘A cyborg world might be about lived social and bodily realities in which people are … not afraid of permanently partial identities and contradictory standpoints’ (Haraway 1985, 13). Under this framework, Frankenstein’s creature is both the essence of creation and definition of destruction; his actions are both pitiable and murderous. ‘The political struggle,’ Haraway asserts, ‘is to see from both perspectives at once because each reveals both dominations and possibilities unimaginable from the other vantage point’ (1985, 13). The crux, according to Haraway, is that a cyborg identity disrupts notions of purity and rebirth. Unlike Dr Frankenstein’s creation, who looks to his ‘father,’ Frankenstein, for salvation through the Edenesque creation of a mate (Haraway 1985, 9), the cyborg is concerned with regeneration. Like the salamander regrowing its tail, cyborgs regrow and restore ‘function with the constant possibility of twinning or other odd topographical productions at the site of former injury’ (Haraway 1985, 17). Here, it is worth emphasising the site of the regeneration: it is the site of the wound, of the loss. A cyborg dream understands the impossibility of a ‘clean slate’ or rebirth; it does not require an erasure of historical trauma. Instead, it grows from that site. (Or, at the very least, finds a way to patch it over—a healing that has no obligation to look or ‘return to’ an imagined ‘normal.’)

Haraway’s vision of mutancy and regeneration offers powerful metaphors in the face of our current totalising narratives: in the cyborg world, we are all mutant. As such, we can collectively channel our energy towards the site of the wound—the mutant virus and its damage—and simultaneously see it not as Other, but as part of our own creation, a product of our deforestation, globalisation and climate change. When the virus spreads through our population and gives rise to its own, mutating, evolving, populations within us, *we—us humans—*create the next generation of the virus. We serve as both host and stranger, both self and other, as the mutants form inside us and spread among us. In the same way that a close reading of *Frankenstein* allows the reader to see the creature as both Victor’s curse and his responsibility, we too can see the newest COVID-19 mutants as both a direct product of our collective actions and as a site to be acted on.

In the cyborg feminist structure, dissolving frameworks of dominance is paramount. These frameworks, in which power is exercised over an Other, take the form of dualisms: ‘self/other, mind/body, culture/nature, male/female, civilized/primitive, reality/appearance, whole/part, agent/resource, maker/made, active/passive, right/wrong, truth/illusion, total/partial, God/man’ (Haraway 1985, 35). Cyborg feminism, however, offers ‘a way out of the maze of dualisms in which we have explained our bodies and our tools to ourselves’ (Haraway 1985, 39). As evidenced by Wald’s study of the outbreak narrative, the movement of bodies, ideas and disease is inevitable, and our reactions to contagion can reflect anything from our worst nightmare to our best daydreams—and everything in between. The current mythologising of the omicron variant as the *Frankenstein* creature forces us to reckon with the confusion of figure and reference. It is a confusion that bears political, social and policy consequences, from global vaccine funding to local mask ordinances. As Haraway explains, ‘There is a myth system waiting to become a political language to ground one way of looking at science and technology and challenging the informatics of domination’ (1985, 38).

## Trajectories of the Frankenstenian myth to describe COVID-19

**Figure 2:**
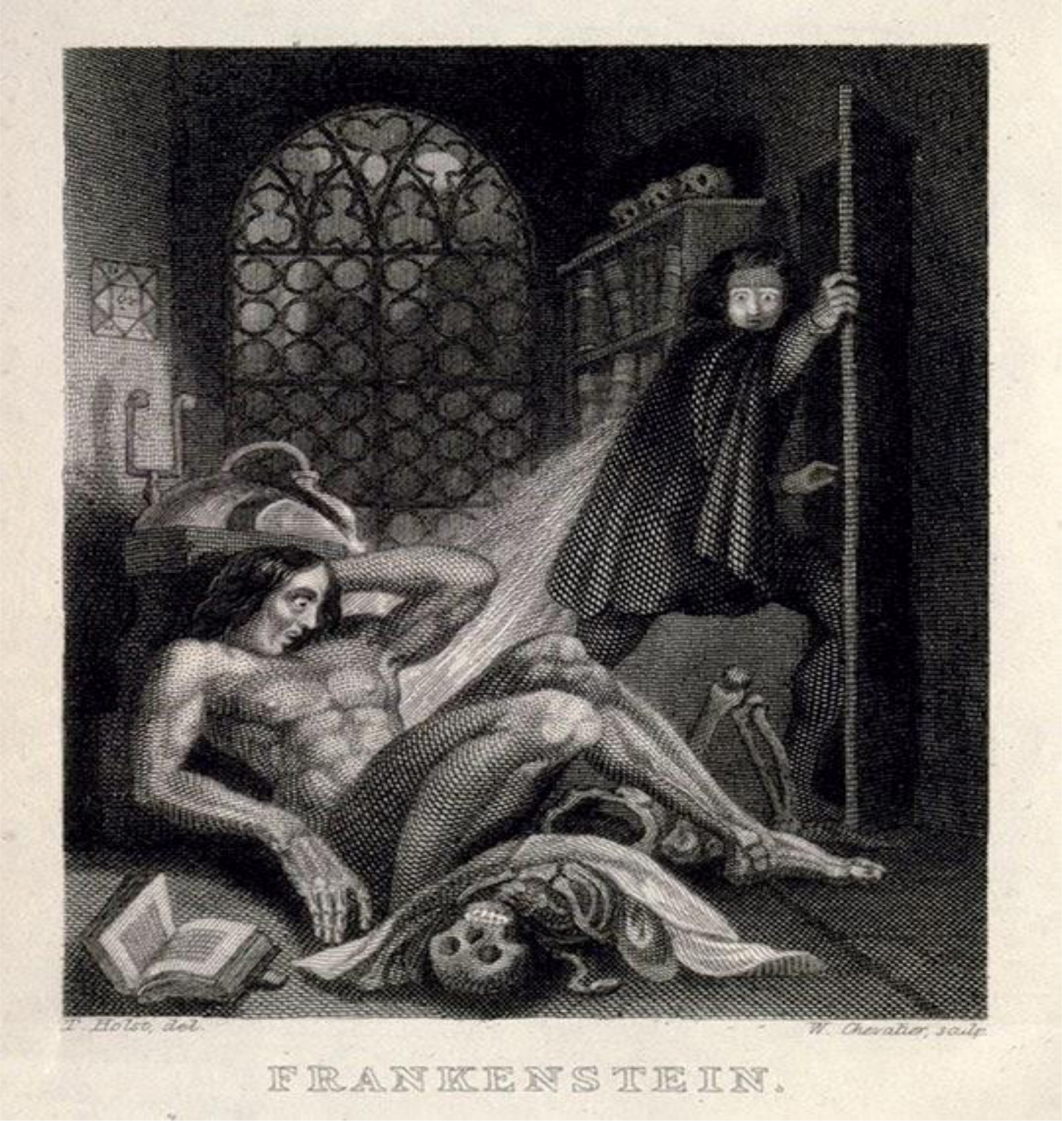
Frontispiece to Frankenstein, 1831 (adapted from Holst 1831).

**Figure 3:**
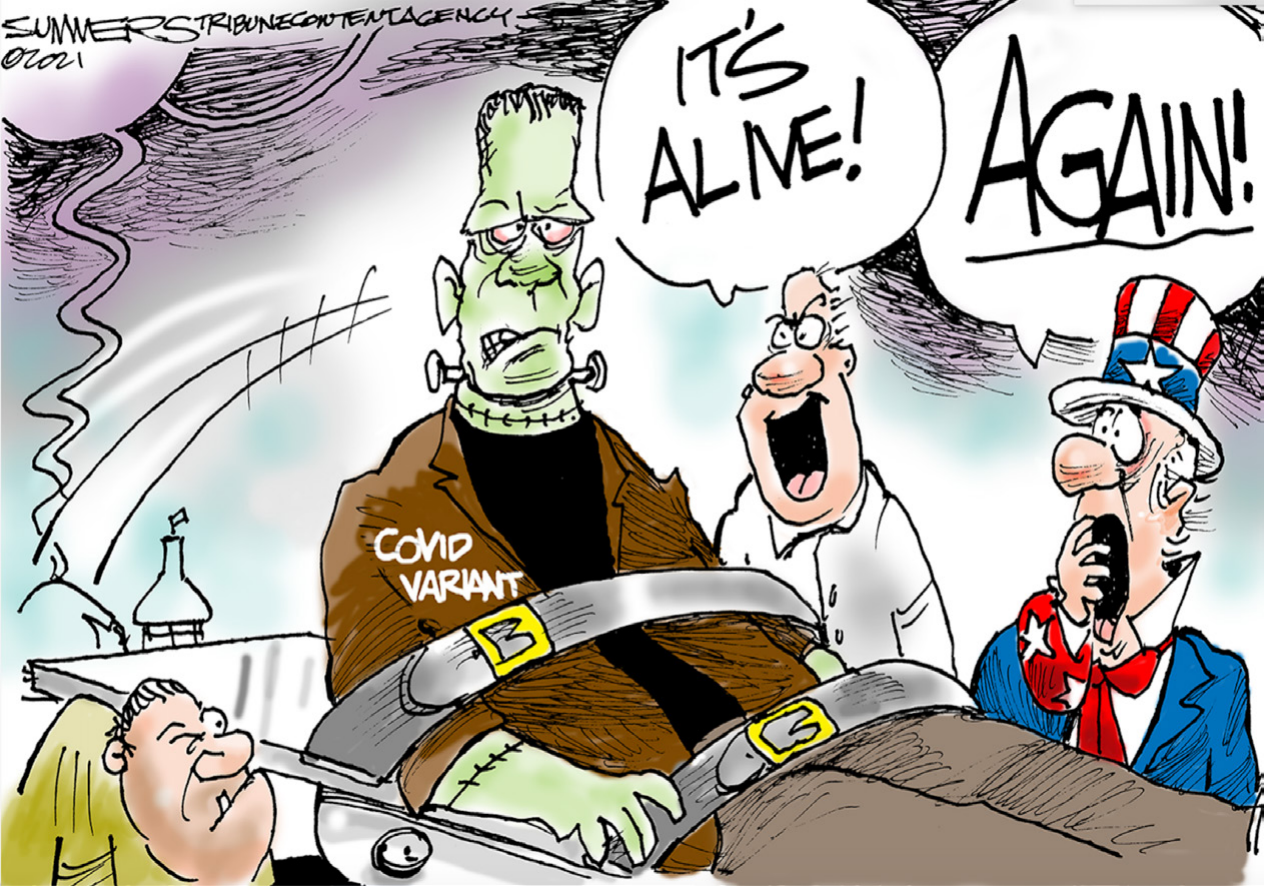
Political cartoon depicting COVID variants, 1 August 2021 (adapted from Summers 2021).

Such political and social language feels distant, something yet to come. But, the COVID-19 pandemic has shown us that this isn’t true. We have been, and remain, in an ever-evolving maze of dualisms of dominance. Take, for instance, the dualism of culture/nature. The tension between the two asks us to delineate the preventable from the inevitable, that which we are actively creating and that which exists beyond us. Culture comes to define us against the backdrop of nature. But, looking to two depictions of the Frankenstein narrative, one the original illustrations that accompanied the release of Mary Shelley’s *Frankenstein* (figure 2) and the other from a political cartoon published in *The Week* in 2021 (figure 3), shows that efforts to separate ‘us’, our culture or humanity, from the viral stories used to reflect nature are futile and disillusioned.

In the classic book cover, the image of Victor Frankenstein fleeing the scene of his creation’s animation represents the genesis of the creature’s othering; it operates within the narrative tradition of creating a being, separate from an ‘us’ (humans), to be feared. In this image, we see the creature sprawled out on the floor, while a divine light shines on their body, suggesting supernatural origin as well as a gesture towards the notion of regeneration. The shocked expression on the creature’s face directs the reader’s attention to Victor Frankenstein. He is depicted in motion, already fleeing the scene. Dr Frankenstein’s partially cloaked face is of special interest: both protected from the creature and protected from our gaze. We bear witness to his act and his shame, his look of horror foreshadowing his desire to conceal his creation. But the divine light reflecting from his creation’s body illuminates Victor Frankenstein’s face, despite his best efforts to shield and flee. Thus, man’s engagement with science serves a revelatory purpose: we see the human, Victor, more clearly, more deeply in this interaction than were we to observe the two characters separately. In this moment, man and ‘mutant’ illuminate one another. Such illumination suggests a mutuality in knowing, and here one can consider the implications of co-recognition between maker and mutant in light of Wald and Haraway: human and ‘mutant’ are irretrievably bound to one another, sharing the narrative frame, even as one attempts to flee, closing the door to contain. This image shows us that even from its inception, the myth of Frankenstein intertwines the concepts of creator and creation, of culture and nature. We do not live separately from the virus, neither literally nor metaphorically; just as our bodies are the catalysts of viral replication and, therefore, evolution and mutagenesis, we are the catalysts of our viral stories.

When we tell outbreak and viral narratives that emphasise perceived distance between us (humans) and nature (viruses), we reinforce our harmful predisposition to create a militant Other. Such is the case with the political cartoon from *The Week* that was published in August 2021, just months before the Omicron variant surfaced. The cartoon references the 1931 film version of Frankenstein starring Boris Karloff, in which the trope of the mad scientist who screams, ‘It’s alive!’ is born. In this version, Uncle Sam, representing the US government and American citizenship, stands aghast, in contrast with the scientist’s fascination and ecstasy. The label VARIANT (read: mutant) on the creature’s jacket leads to the politician’s scream of ‘AGAIN!’—yet another variant has emerged; containment has not prevented the revival of the viral threat. The cartoon depicts a cycle of mutability, an ever-changing and emerging sphere of communicable disease: variants will emerge, and they will be nested within our larger COVID-19 narrative. In this cartoon, science and governance are separate entities with different motivations and reactions to events, forming additional dualisms of dominance: science/governance, science/virus, governance/virus. This fragmented view of outbreak dilutes and transfers responsibility away from the body. The regimented separation of science, governance and creature in the cartoon communicates a disjointed reality; the body of the viewer is not one with the virus nor with the science behind it.

The viral stories we tell define us, and, as microbiologist, queer activist and writer, Joseph Osmundson writes, ‘viral stories will change, their center will not hold’ (2022, 36). These stories have defined us, do define us and will define us. From the inception of Frankenstein up to the present, the myth has been told and retold, shifting, transforming, mutating with each iteration. We have then and now, the future comes next. ‘The virus. Our body. Our biomedicine. Only through the three together can meaning be made; that meaning will shift over time as the virus, our bodies, and biomedicine do change’ (Osmundson 2022, 26). Rather than continue with the failing logic of separation and fragmentation, we can reconfigure the body and its relationship to a viral reality.

## Conclusion

Creating, investing in and perpetuating outbreak narratives informed by cyborg feminism, wherein artificially drawn boundaries are acknowledged and dissolved, may be one way forward. ‘As long as we live, viruses will almost-live alongside us’, Osmundson writes (2022, 60). ‘This is not to minimise the lives lost to HIV or COVID-19 or to any other virus’, but rather to acknowledge a basic fact: ‘We live on a viral planet. They were here first. We are their guests, not hosts. That is a viral story worth telling’ (Osmundson 2022, 60).

What might it look like, feel like and indeed even *be* like to live in a world in which the metaphor of a mutant as an Other is not the foundation of messaging and policy—is not used to drive ineffective, fear-based, racist responses to emerging viral variants? It is not enough to simply pose this question: it is incumbent on us—individuals and the institutions we form—to respond with serious, thoughtful imaginative labour.

Certainly, one place to start is language. Public health crises tend to introduce new phrases and acronyms into the lexicon. One need not look further than the new and omnipresent ‘social distancing’ phrase or the now widely known abbreviation of ‘PPE’ (personal protective equipment) to see how public health crises bring new rhetoric. What other terms might we create? What kind of conversations and policies might we construct if we extended our ‘pandemic bubbles’ to include vaccine access for all?

Such imagining may lead us to the material issue of funding for COVID-19 Vaccines Global Access, known as COVAX, a public health, multipartner, global delivery programme of COVID-19 vaccines. It may steer us away from accepting a world in which the pandemic is ‘over’ for some yet rages on for others in ‘Other’ places. As of the authors’ writing, COVAX remains severely underfunded, despite the reality that global vaccine distribution is essential to the health of a global body politic. After all, with widespread gaps in immunisation, viral replication persists, yielding new variants that can evade vaccine-induced immunity.

These imaginings may also provide inroads for new ways of teaching and conceiving of public health. As the organisation Public Health Liberation explains, ‘Language Legacy’, is ‘the tendency of dominant culture to keep in place those traits in the language that maintain power and dominance over the social structures of the society’. This includes and relies on, the report continues, ‘symbol systems—verbal and nonverbal’ (Health Equity and Liberation 2022, 16). As we have demonstrated, metaphor and its associations—particularly those around Frankenstein (regardless of how true they are to the text), mutants, Othering and viruses—are essential to maintaining and perpetuating existing power structures. How might public health practitioners and educators include accounts of and responses to public health injustice and inequity in their work? This is a question many in the field are taking up—and one that demands more resources and attention.

And in considering how it would *feel* to live in a world in which the binaries of ‘human/virus’ and ‘self/other’ are seen as false, one must consider the role of horror as an affect. Osmundon reminds us, ‘Horror grabs our attention, even when it’s a lie, even when it’s a metaphor’ (2022, 60). Indeed the very backbone of the *Frankenstein* novel is the mixture of horror and science fiction—it is, one could argue, a mutant of the two. Horror plays a crucial role in capturing attention and is a powerful mechanism for propagating myth and its metaphors. ‘HIV, COVID-19, Ebola, and rabies can kill,’ writes Osmundson, ‘The problem wasn’t illness. The problem never is. Illness is a fact of life. The problem is our inability to provide care to all’.

A litmus test, then, of our metaphors may be: Does it move us in the direction of extending care? Does it provide imaginative possibilities that expose, rather than reinforce, false boundaries and binaries? And what is the role of horror in the narrative schema?

If COVID-19 has taught us anything, it is that there will be more variants and future chapters of the pandemic narrative. When the next variant emerges, will we default to existing, oppressive frameworks? Or will we find ways to regenerate, embracing our own mutancy and imagine more equitable and effective pathways to respond?

## Data Availability

There are no data in this work.
